# Supplementary material for: The regulatory landscape of the human HPF1- and ARH3-dependent ADP-ribosylome
Source: Nat Commun. 2021 Oct 8;12:5893. doi: 10.1038/s41467-021-26172-4 (PMC8501107; doi:10.1038/s41467-021-26172-4)
Supplement: Supplementary file 1 — Supplementary Information [file 41467_2021_26172_MOESM1_ESM.pdf]

SUPPLEMENTARY INFORMATION

The regulatory landscape of the human HPF1- and ARH3-dependent ADP-ribosylome

Hendriks & Buch-Larsen et al.

Supplementary Figures 1–7

Supplementary Table 1

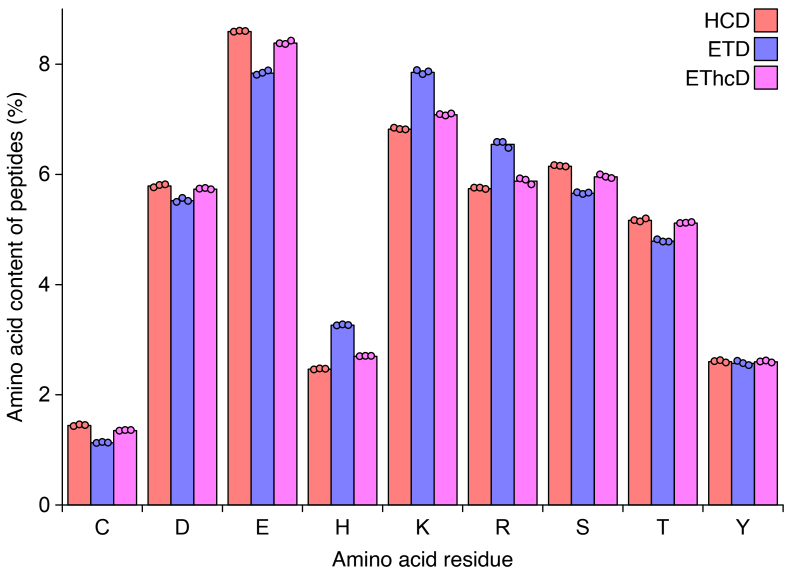

HCD
ETD
EThcD
Amino acid content of peptides (%)
0
2
4
6
8
C
D
E
H
K
R
S
T
Y
Amino acid residue

Supplementary Figure 1. Evaluation of HCD, ETD, and EThcD fragmentation. Distribution of ADPr-reactive amino acids as detected using HCD, ETD, and EThcD fragmentation, by measuring HeLa total cell lysate. Data are presented as mean values, *n*=3 technical replicate measurements from the same HeLa total cell lysate. Source data are provided as a Source Data file.

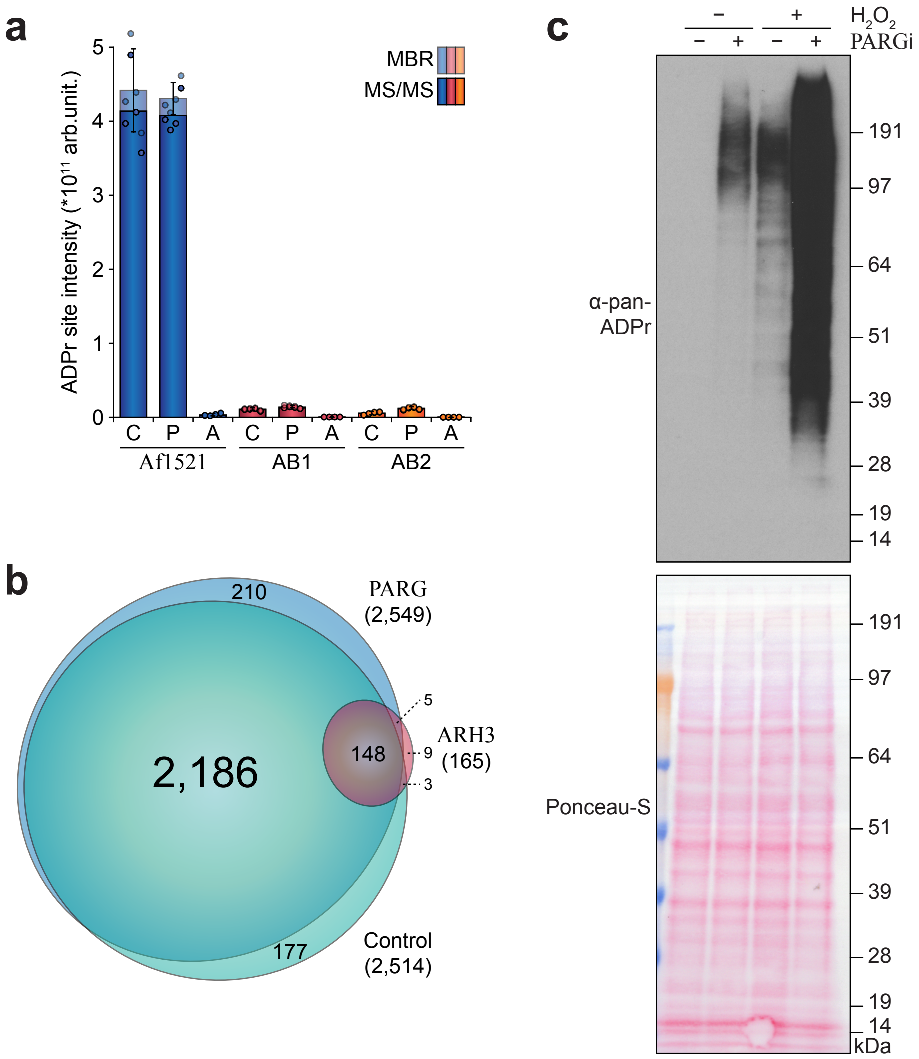
a
b
c
MBR
MS/MS
ADPr site intensity (*10¹¹ arb.unit.)
C
P
A
Af1521
AB1
AB2
210
PARG
(2,549)
2,186
148
5
9
3
ARH3
(165)
177
Control
(2,514)
H2O2
PARGi
α-pan-
ADPr
Ponceau-S
191
97
64
51
39
28
19
14
kDa

Supplementary Figure 2. Evaluation of different erasers and enrichers for the purification of ADPr. (A) Overview of the ADPr site abundance across experimental conditions. Data are presented as mean values +/− SD, n=4 purification replicates. “C”; control, “P”; PARG, “A”; ARH3, “MBR”; matching between runs. (B) Scaled Venn diagram depicting overlap between ADPr sites in Af1521-enriched samples. (C) Immunoblot analysis showing that PARGi treatment of HeLa cells results in a large accumulation of ADPr signal in the context of H_2O_2-treatment. Treatment with PARGi was performed for 2 days at 25 μM, and treatment with H_2O_2 was performed for 10 min at 1 mM. Ponceau-S analysis serves as loading control. This experiment was performed as two independent biological replicates, with similar results. Source data are provided as a Source Data file.

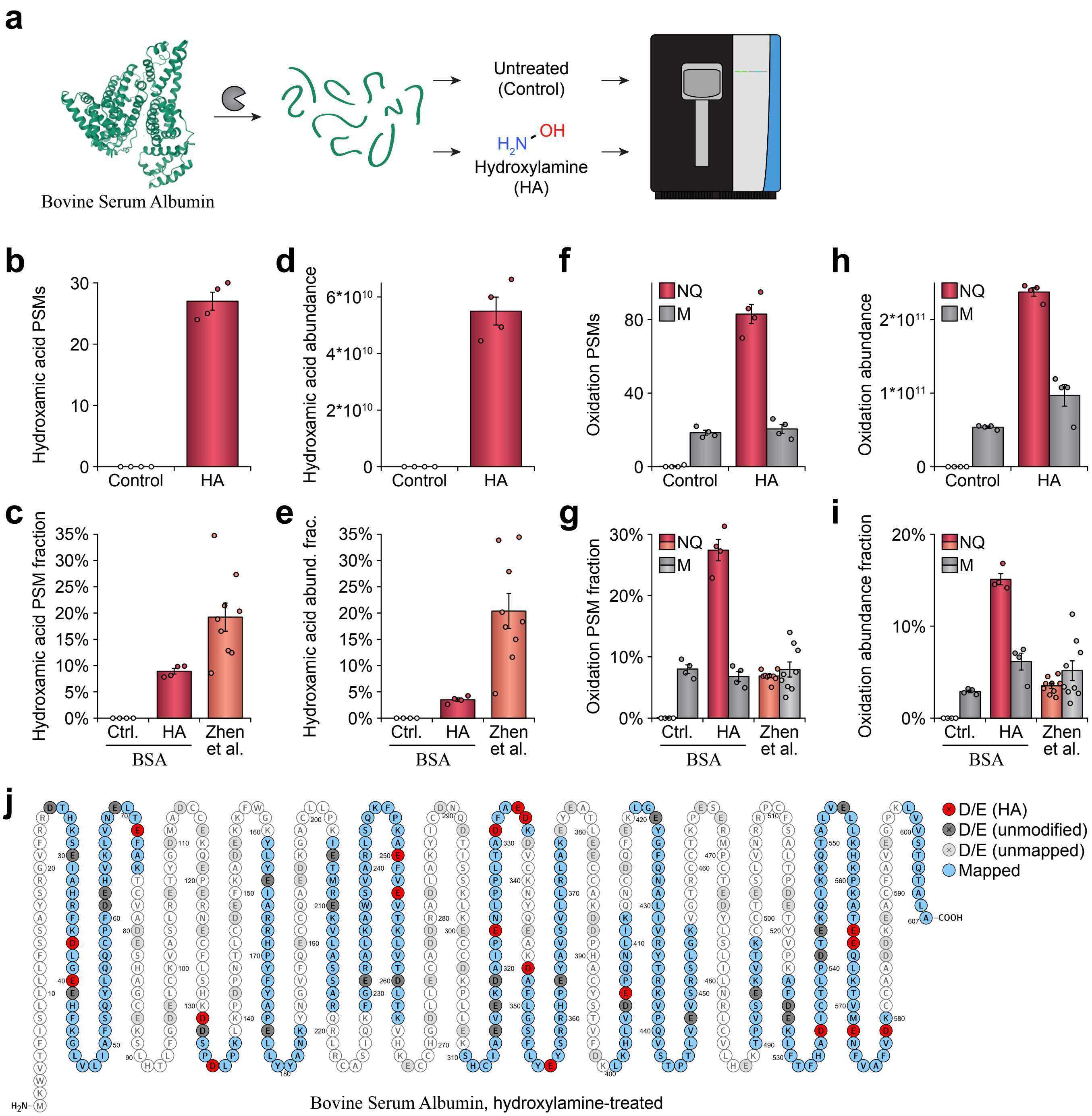
a
Bovine Serum Albumin
Untreated
(Control)
H_2N-OH
Hydroxylamine
(HA)
b
Hydroxamic acid PSMs
30
20
10
0
Control
HA
d
Hydroxamic acid abundance
6*10^10
4*10^10
2*10^10
0
Control
HA
f
Oxidation PSMs
NQ
M
80
40
0
Control
HA
h
Oxidation abundance
NQ
M
2*10^11
1*10^11
0
Control
HA
c
Hydroxamic acid PSM fraction
35%
30%
25%
20%
15%
10%
5%
0%
Ctrl.
HA
Zhen et al.
BSA
e
Hydroxamic acid abund. frac.
35%
30%
25%
20%
15%
10%
5%
0%
Ctrl.
HA
Zhen et al.
BSA
g
Oxidation PSM fraction
NQ
M
30%
20%
10%
0%
Ctrl.
HA
Zhen et al.
BSA
i
Oxidation abundance fraction
NQ
M
20%
10%
0%
Ctrl.
HA
Zhen et al.
BSA
j
D/E (HA)
D/E (unmodified)
D/E (unmapped)
Mapped
H_2N-
-COOH
Bovine Serum Albumin, hydroxylamine-treated

Supplementary Figure 3. Investigation of hydroxylamine-induced chemical artefacts. (A) Overview of the experimental design. (B) Visualization of the number of peptide-spectrum-matches (PSMs) identified with hydroxamic acid on aspartate and glutamate residues. *n*=4 technical replicates. Data are presented as mean values +/− SEM. (C) As **B**, but showing the fraction of modified PSMs in relation to all PSMs. Additionally, breast cancer cell line ADPr proteomics data from Zhen et al.[1], was re-processed and inspected for hydroxamic acid on aspartate and glutamate residues, using computational settings identical to those used for the BSA data. *n*=9 distinct cell lines, as derived from Zhen et al. (D) Visualization of the abundance (intensity, arb. unit) of peptides modified with hydroxamic acid. *n*=4 technical replicates. Data are presented as mean values +/− SEM. (E) As **D**, but showing the fraction of modified abundance in relation to total abundance, with inclusion of the re-processed data from Zhen et al. (as **C**). (F) Visualization of the number of PSMs identified with oxidation of asparagine, glutamine, and methionine residues. *n*=4 technical replicates. Data are presented as mean values +/− SEM. (G) As **F**, but showing the fraction of modified PSMs in relation to all PSMs, with inclusion of the re-processed data from Zhen et al. (as **C**). (H) Visualization of the abundance (intensity, arb. unit) of peptides modified with oxidation. *n*=4 technical replicates. Data are presented as mean values +/− SEM. (I) As **H**, but showing the fraction of modified abundance in relation to total abundance, with inclusion of the re-processed data from Zhen et al. (as **C**). (J) Schematic representation of BSA, visualizing which part of the sequence was detected (in blue). Aspartate and glutamate residues which were modified by hydroxamic acid after HA treatment are indicated in red, non-modified residues are indicated in grey. Source data are provided as a Source Data file.

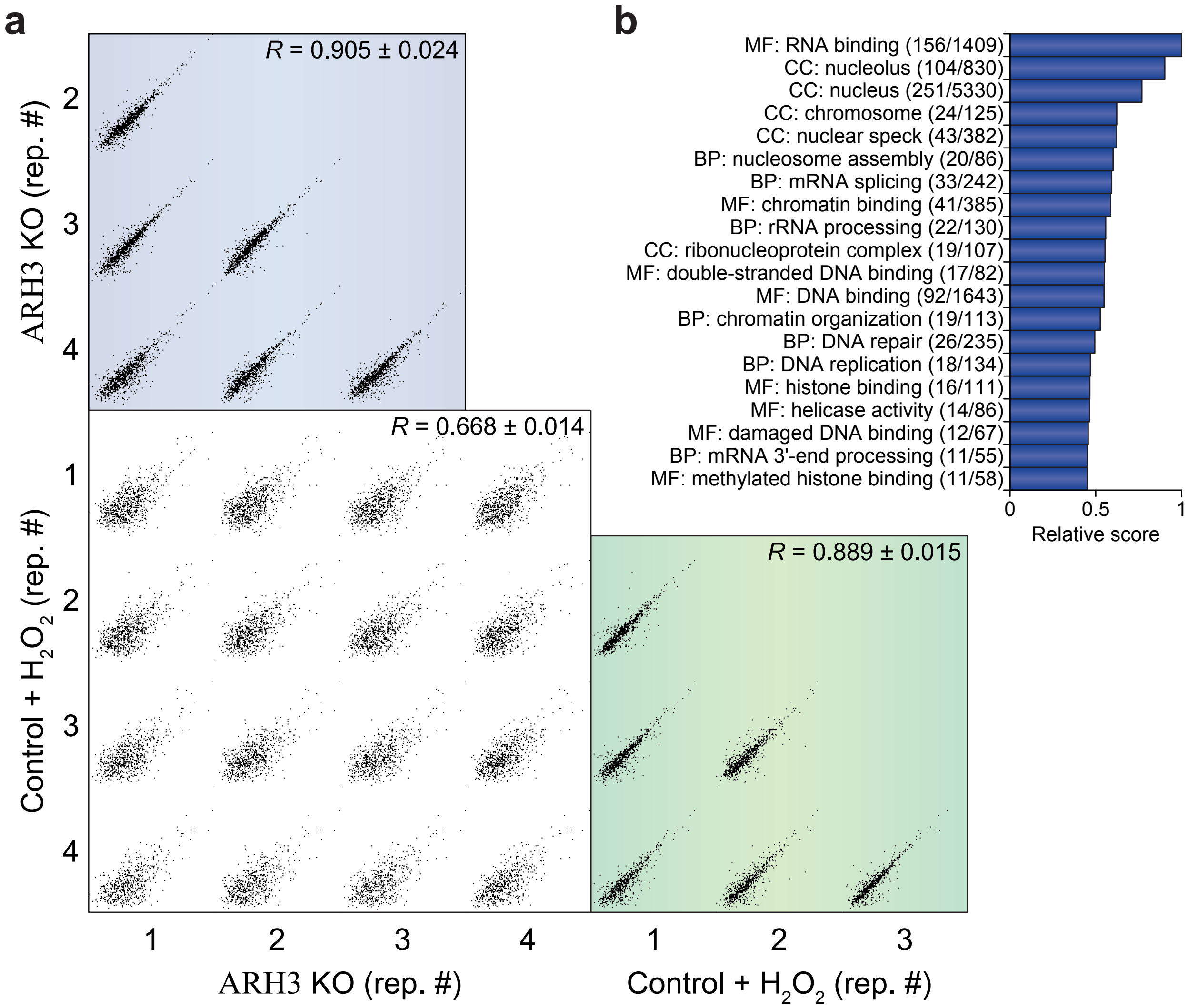
a
R = 0.905 ± 0.024
ARH3 KO (rep. #)
2
3
4
R = 0.668 ± 0.014
Control + H_2O_2 (rep. #)
1
2
3
4
R = 0.889 ± 0.015
1 2 3 4
ARH3 KO (rep. #)
1 2 3
Control + H_2O_2 (rep. #)
b
MF: RNA binding (156/1409)
CC: nucleolus (104/830)
CC: nucleus (251/5330)
CC: chromosome (24/125)
CC: nuclear speck (43/382)
BP: nucleosome assembly (20/86)
BP: mRNA splicing (33/242)
MF: chromatin binding (41/385)
BP: rRNA processing (22/130)
CC: ribonucleoprotein complex (19/107)
MF: double-stranded DNA binding (17/82)
MF: DNA binding (92/1643)
BP: chromatin organization (19/113)
BP: DNA repair (26/235)
BP: DNA replication (18/134)
MF: histone binding (16/111)
MF: helicase activity (14/86)
MF: damaged DNA binding (12/67)
BP: mRNA 3'-end processing (11/55)
MF: methylated histone binding (11/58)
0 0.5 1
Relative score

Supplementary Figure 4. The ADP-ribosylome in HPF1 and ARH3 knockout cells. (A) ‘B-2 bomber plot’ visualizing the correlation of ADPr site intensities between all H_2O_2-treated control samples and untreated ARH3 KO samples. Numbers indicate Pearson correlation ± SD. (B) Term enrichment analysis using Gene Ontology annotations, comparing ADPr target proteins detected in both H_2O_2-treated control cells and untreated ARH3 KO cells, to the total proteome. Relative score is based on multiplication of logarithms derived from the enrichment ratio and the q-value. Terms were significant with q<0.02, as determined through Fisher Exact Testing with Benjamini-Hochberg correction. “BP”; biological process, “CC”; cellular compartment, “MF”; molecular function. Source data are provided as a Source Data file.

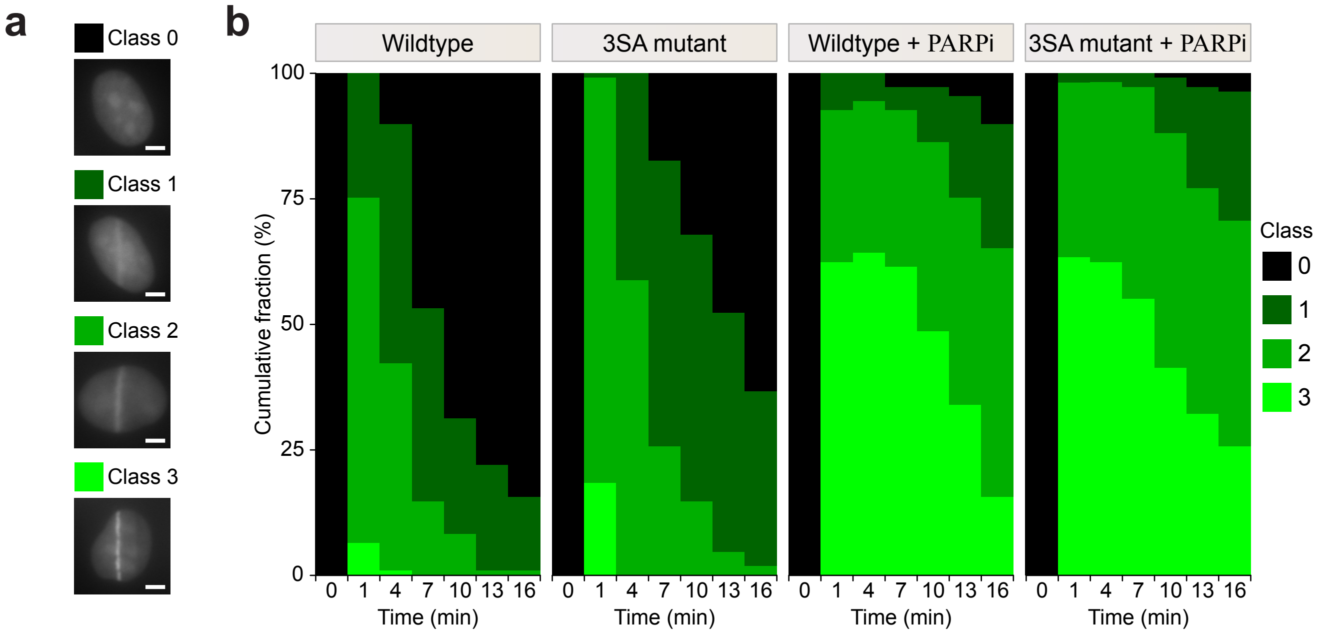
a
Class 0
Class 1
Class 2
Class 3
b
Wildtype
3SA mutant
Wildtype + PARPi
3SA mutant + PARPi
Cumulative fraction (%)
100
75
50
25
0
0 1 4 7 10 13 16
Time (min)
Class
0
1
2
3

Supplementary Figure 5. Analysis of PARP1 recruitment to DNA damage. (A) Representative live cell microscopy images, visualizing stratification classes. “0”; laser stripe not visible, “1”; laser stripe barely visible, “2”; laser stripe moderately visible, “3”; laser stripe strongly visible. The scale bar represents 5 µm. (B) Quantification of recruitment of GFP-PARP1 to damage stripes, as monitored via all stratification classes. PARP1 kinetics were significantly different between WT and 3SA mutant in the absence of PARPi, at $p<0.001$ for T=1,4,7,10,13, and at $p<0.05$ for T=16. In presence of PARPi, a significant difference was observed between WT and 3SA at T=7, at $p<0.05$, with no significant differences at other time points. Significance determined via Chi-squared testing, $n=109$ cells per condition.

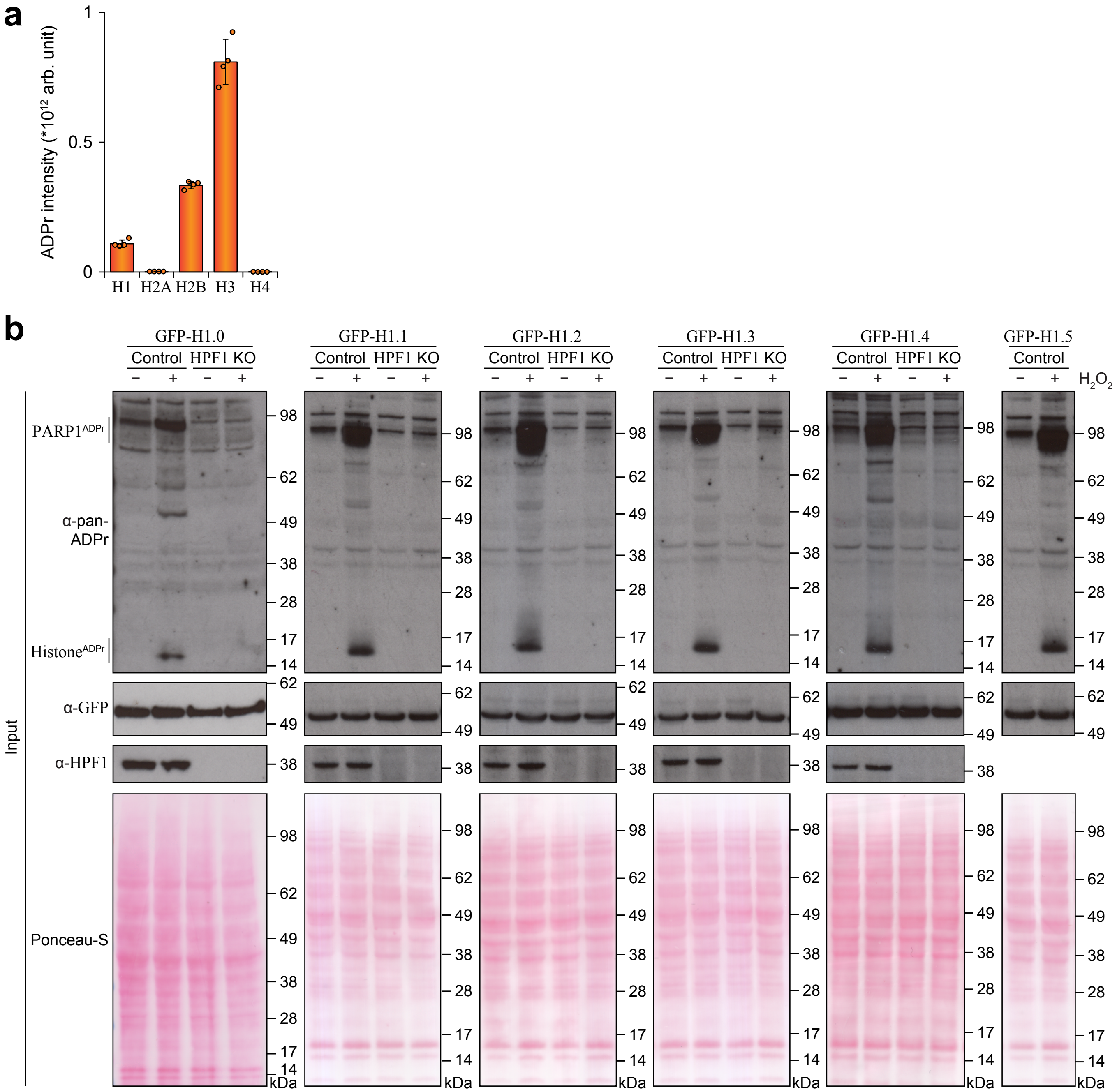
a
ADPr intensity (*10^12 arb. unit)
1
0.5
0
H1
H2A
H2B
H3
H4
b
GFP-H1.0
GFP-H1.1
GFP-H1.2
GFP-H1.3
GFP-H1.4
GFP-H1.5
Control
HPF1 KO
H2O2
Input
PARP1ADPr
α-pan-ADPr
HistoneADPr
α-GFP
α-HPF1
Ponceau-S
98
62
49
38
28
17
14
kDa

Supplementary Figure 6. ADPr modification of histone family members. (A) Overview of the fraction of ADPr residing on histones, merged for all experimental conditions. *n*=4 cell culture replicates, data are presented as mean values +/− SD. (B) Input and loading control immunoblots and Ponceau-S analysis corresponding to Figure 4D. This experiment was performed as two independent biological replicates, with similar results. Source data are provided as a Source Data file.

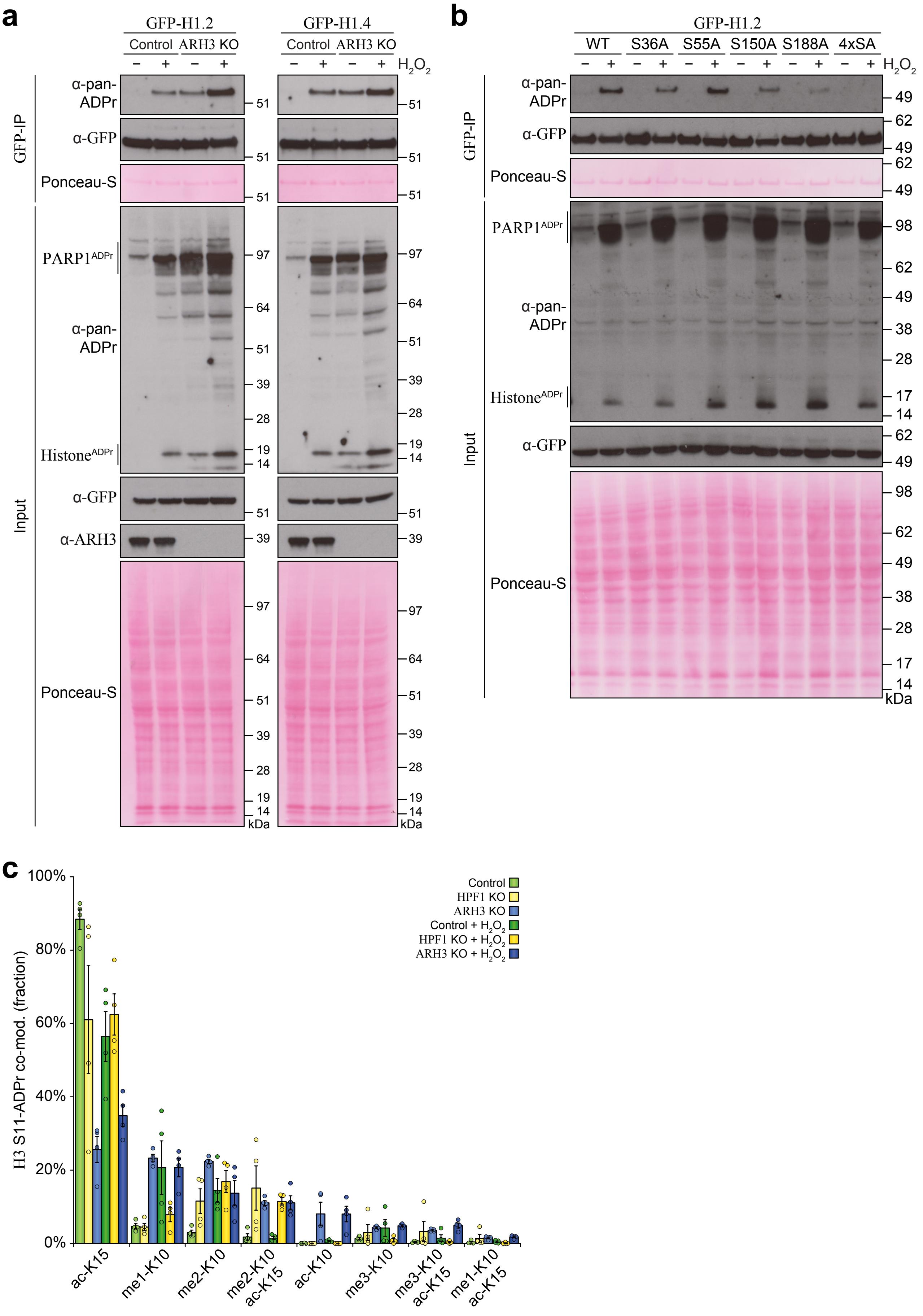
a
GFP-H1.2
GFP-H1.4
Control
ARH3 KO
H2O2
GFP-IP
α-pan-ADPr
α-GFP
Ponceau-S
Input
PARP1ADPr
HistoneADPr
α-ARH3
kDa
97
64
51
39
28
19
14
b
WT
S36A
S55A
S150A
S188A
4xSA
98
62
49
38
17
c
H3 S11-ADPr co-mod. (fraction)
100%
80%
60%
40%
20%
0%
Control
HPF1 KO
ARH3 KO
Control + H2O2
HPF1 KO + H2O2
ARH3 KO + H2O2
ac-K15
me1-K10
me2-K10
me2-K10 ac-K15
ac-K10
me3-K10
me3-K10 ac-K15
me1-K10 ac-K15

Supplementary Figure 7. Validation of Histone H1.2 and H1.4 serine ADPr. (A) Immunoblot experiments performed in wild-type or ARH3 KO HEK293T cells, transiently transfected with the indicated GFP-tagged histones for 24 h. H_2O_2 treatment was performed at 2 mM for 10 min, after which cells were lysed and GFP-IP was performed. Ponceau-S analysis serves as a loading control. This experiment was performed as two independent biological replicates, with similar results. (B) As **A**, but analyzing serine-to-alanine substitution mutants of GFP-tagged Histone H1.2, using wild-type HEK293T cells. "4xSA"; mutant with all four serine-to-alanine mutations. (C) Visualization of the fractional abundance of S11-ADPr co-modifications occurring on Histone H3. n=4 cell culture replicates, data are presented as mean values +/− SEM. Source data are provided as a Source Data file.

Primer name	Primer sequence
H1.2 S36A forward	5'-CGGGGGACCGGCCGCCTTACGAG-3'
H1.2 S36A reverse	5'-CTCGTAAGGCGGCCGGTCCCCCG-3'
H1.2 S55A forward	5'-CTCTAAAGAGCGTGCCGGAGTTTCTCTG-3'
H1.2 S55A reverse	5'-CAGAGAAACTCCGGCACGCTCTTTAGAG-3'
H1.2 S150A forward	5'-TCGGTGTTTTCTTAGCGGCCTTCTTCGGAGTTGCGC-3'
H1.2 S150A reverse	5'-GCGCAACTCCGAAGAAGGCCGCTAAGAAAACACCGA-3'
H1.2 S188A forward	5'-CGAAGCCCAAGAAAGCTGCCAAAGCTGCTGCTAAGGC-3'
H1.2 S188A reverse	5'-GCCTTAGCAGCAGCTTTGGCAGCTTTCTTGGGCTTCG-3'

Supplementary Table 1. A list of all primers used in this study.

Supplementary References

1. Zhen Y, Zhang Y, Yu Y. A Cell-Line-Specific Atlas of PARP-Mediated Protein Asp/Glu-ADP-Ribosylation in Breast Cancer. *Cell Rep* **21**, 2326-2337 (2017).
