# Supplementary material for: The regulatory landscape of the human HPF1- and ARH3-dependent ADP-ribosylome
Source: Nat Commun. 2021 Oct 8;12:5893. doi: 10.1038/s41467-021-26172-4 (PMC8501107; doi:10.1038/s41467-021-26172-4)
Supplement: Supplementary file 3 — Description of Additional Supplementary Files [file 41467_2021_26172_MOESM3_ESM.docx]

**Description of Additional Supplementary Files**

File name: Supplementary Data 1

Description: Overview of ADPr sites identified *in vitro*. A list of all ADPr sites identified in the *in vitro* experiments, complete with qualitative and quantitative information. *n*=3 technical replicates.

File name: Supplementary Data 2

Description: Overview of ADPr sites identified in Af1521 and antibody experiments. A list of all 2,758 unique human ADPr sites identified in the Af1521 and pan-ADPr antibody comparison experiments, complete with qualitative and quantitative information. *n*=4 cell culture replicates.

File name: Supplementary Data 3

Description: Overview of hydroxamic acid and oxidation events detected in hydroxylamine-treated BSA. A list of all hydroxamic acid (DE) sites found on BSA after mock or hydroxylamine treatment, and an overview of all MaxQuant evidence for hydroxamic acid (DEM), oxidation (MNQ), and deamidation (NQ) events. *n*=4 technical replicates.

File name: Supplementary Data 4

Description: Overview of hydroxamic acid and oxidation events detected in re-processed breast cancer cell line data. An overview of all MaxQuant evidence for hydroxamic acid (DEM), oxidation (MNQ), and deamidation (NQ) events, found in re-processed MS raw data. Original MS raw data has been previously described^31^. *n*=9 different cell lines.

File name: Supplementary Data 5

Description: Overview of ADPr sites identified in boronic acid enrichment experiments. A list of all human ADPr sites identified in the boronic acid enrichment experiments, complete with qualitative and quantitative information. *n*=4 cell culture replicates.

File name: Supplementary Data 6

Description: Overview of ADPr sites identified in HPF1 KO and ARH3 KO experiments. A list of all 1,596 unique human ADPr sites identified in the HPF1 KO and ARH3 KO experiments, complete with qualitative and quantitative information. *n*=4 cell culture replicates.

File name: Supplementary Data 7

Description: Overview of ADPr target proteins identified in HPF1 KO and ARH3 KO experiments. A list of all 799 unique human ADPr target proteins identified in the HPF1 KO and ARH3 KO experiments, complete with qualitative and quantitative information. *n*=4 cell culture replicates.

File name: Supplementary Data 8

Description: Overview of co-modified ADPr peptides and sites identified in HPF1 KO and ARH3 KO experiments. A list of all 146 unique human ADPr-co-modified sites identified in the HPF1 KO and ARH3 KO experiments, complete with qualitative and quantitative information. *n*=4 cell culture replicates.
