# Supplementary material for: The regulatory landscape of the human HPF1- and ARH3-dependent ADP-ribosylome
Source: Nat Commun. 2021 Oct 8;12:5893. doi: 10.1038/s41467-021-26172-4 (PMC8501107; doi:10.1038/s41467-021-26172-4)
Supplement: Supplementary file 12 — Reporting Summary [file 41467_2021_26172_MOESM12_ESM.pdf]

nature portfolio

Corresponding author(s): Michael Lund Nielsen

Last updated by author(s): Aug 30, 2021

Reporting Summary

Nature Portfolio wishes to improve the reproducibility of the work that we publish. This form provides structure for consistency and transparency in reporting. For further information on Nature Portfolio policies, see our Editorial Policies and the Editorial Policy Checklist.

Statistics

For all statistical analyses, confirm that the following items are present in the figure legend, table legend, main text, or Methods section.

n/a	Confirmed	
☐	☒	The exact sample size (n) for each experimental group/condition, given as a discrete number and unit of measurement
☐	☒	A statement on whether measurements were taken from distinct samples or whether the same sample was measured repeatedly
☐	☒	The statistical test(s) used AND whether they are one- or two-sided<br>*Only common tests should be described solely by name; describe more complex techniques in the Methods section.*
☐	☒	A description of all covariates tested
☐	☒	A description of any assumptions or corrections, such as tests of normality and adjustment for multiple comparisons
☐	☒	A full description of the statistical parameters including central tendency (e.g. means) or other basic estimates (e.g. regression coefficient) AND variation (e.g. standard deviation) or associated estimates of uncertainty (e.g. confidence intervals)
☐	☒	For null hypothesis testing, the test statistic (e.g. F, t, r) with confidence intervals, effect sizes, degrees of freedom and P value noted<br>*Give P values as exact values whenever suitable.*
☒	☐	For Bayesian analysis, information on the choice of priors and Markov chain Monte Carlo settings
☒	☐	For hierarchical and complex designs, identification of the appropriate level for tests and full reporting of outcomes
☐	☒	Estimates of effect sizes (e.g. Cohen's d, Pearson's r), indicating how they were calculated

Our web collection on statistics for biologists contains articles on many of the points above.

Software and code

Policy information about availability of computer code

Data collection	No custom software was used. All software used in the manuscript is publicly available and described in the methods. Software used: MaxQuant v1.5.3.30, Perseus v1.5.5.3, and iceLogo v1.2.
Data analysis	No custom software was used. All software used in the manuscript is publicly available and described in the methods. Software used: MaxQuant v1.5.3.30, Perseus v1.5.5.3, and iceLogo v1.2.

For manuscripts utilizing custom algorithms or software that are central to the research but not yet described in published literature, software must be made available to editors and reviewers. We strongly encourage code deposition in a community repository (e.g. GitHub). See the Nature Portfolio guidelines for submitting code & software for further information.

Data

Policy information about availability of data

All manuscripts must include a data availability statement. This statement should provide the following information, where applicable:

- Accession codes, unique identifiers, or web links for publicly available datasets
- A description of any restrictions on data availability
- For clinical datasets or third party data, please ensure that the statement adheres to our policy

The mass spectrometry proteomics data generated in this study have been deposited in the ProteomeXchange Consortium via the PRIDE80 partner repository, under accession codes PXD023835 [https://www.ebi.ac.uk/pride/archive/projects/PXD023835] and PXD027504 [https://www.ebi.ac.uk/pride/archive/projects/PXD027504].

All other data generated in this study are provided in the Supplementary Information/Source Data file. Source data are provided with this paper.

The breast cancer cell line proteomics data31 used in this study are available in the Chorus database under accession code 6153436701505083307 [https://chorusproject.org/anonymous/download/experiment/6153436701505083307].

Field-specific reporting

Please select the one below that is the best fit for your research. If you are not sure, read the appropriate sections before making your selection.

- [x] Life sciences
- [ ] Behavioural & social sciences
- [ ] Ecological, evolutionary & environmental sciences

For a reference copy of the document with all sections, see nature.com/documents/nr-reporting-summary-flat.pdf

Life sciences study design

All studies must disclose on these points even when the disclosure is negative.

Sample size	Samples-size calculation was not performed. One MS analysis was performed in technical triplicate. Further MS analyses for all primary experiments was performed in biological quadruplicate (independent cell cultures). The number of cells cultured for each replicate was based on previous reports and observations, in order to gain sufficient depth of sequencing and accurate profiling of low stoichiometry PTMs. Western blot analyses were performed in biological duplicate (independent cell cultures). Microscopy experiments were performed in biological duplicate, and 109 individual cells were investigated from randomly chosen fields of cells spread across the different experiments. The chosen sample sizes are consistent with previous publications, and are common practice in the field (2-4 biological replicates).
Data exclusions	No data was excluded.
Replication	MS analyses were performed in triplicate or quadruplicate. Western blot and microscopy experiments were performed in biological duplicate. All attempts at replication were successful, and the number of replicates are indicated in the figure legends in the manuscript.
Randomization	Samples were not divided into experimental groups, all replicates for all individual experiments were simultaneously prepared, handled, and statistically processed while taking multiple-hypotheses testing into account.
Blinding	All samples relating to each experiment were handled simultaneously. During handling, all samples were numbered and processed in random order to avoid introduction of bias into the samples. During MS data acquisition, samples were clearly labeled (and thus not blinded), which is important to MS experimental design. The performance of the MS instrument drifts over time, and there can be power outages and other factors outside of our control. Therefore, it is important to run samples in an order where the least technical variance is introduced between runs (e.g. control_rep1, treatmentA_rep1, treamtentB_rep1, control_rep2, treatmentA_rep2, treatmentB_rep2, etc.). Further, this lets us account for the limited degree of sample carryover as a result from column carryover.<br><br>All data analysis was performed with unbiased software, in an unsupervised manner, and therefore blinding is not applicable in this context. All MS data is publicly available and may be re-processed and investigated by any external party.

Reporting for specific materials, systems and methods

We require information from authors about some types of materials, experimental systems and methods used in many studies. Here, indicate whether each material, system or method listed is relevant to your study. If you are not sure if a list item applies to your research, read the appropriate section before selecting a response.

Materials & experimental systems

n/a	Involved in the study
☐	☒ Antibodies
☐	☒ Eukaryotic cell lines
☒	☐ Palaeontology and archaeology
☒	☐ Animals and other organisms
☒	☐ Human research participants
☒	☐ Clinical data
☒	☐ Dual use research of concern

Methods

n/a	Involved in the study
☒	☐ ChIP-seq
☒	☐ Flow cytometry
☒	☐ MRI-based neuroimaging

Antibodies

Antibodies used	The following primary antibodies were used for immunoblot analysis in this study, and diluted at 1:1,000 unless stated otherwise. Poly/Mono-ADP Ribose (E6F6A) Rabbit mAb #83732 (CST), PARP1 (46D11) Rabbit mAb #9532 (CST), HPF1 Rabbit pAb HPA043467 (Atlas Antibodies), ADPRHL2 (ARH3) Rabbit pAb HPA027104 (Atlas Antibodies), GAPDH Rabbit pAb ab9485 (Abcam), GFP Mouse mAb clone 7.1 and 13.1 (Roche). Pan-ADPr (MABE1016) Rabbit (Millipore); at 1:1,500, ADPRHL2 (ARH3) Rabbit pAb HPA027104 (Atlas Antibodies); at 1:2,000, GFP (ab290) Rabbit (Abcam); at 1:5,000, custom-made HPF1 antibody was previously described (Bilokapic S, Suskiewicz MJ, Ahel I, Halic M. Bridging of DNA breaks activates PARP2-HPF1 to modify chromatin. Nature 585, 609-613 (2020).)

Secondary antibodies used were Goat-anti-rabbit HRP conjugated secondary antibody (Jackson Immunoresearch, 111-036-045) or Goat-anti-mouse HRP conjugated secondary antibody (Jackson Immunoresearch, 115-036-062), at a concentration of 1:10,000 for 1 h at room temperature.

Validation

Poly/Mono-ADP Ribose (E6F6A) Rabbit mAb #83732 (CST) validation stated on supplier's website: https://www.cellsignal.com/products/primary-antibodies/poly-mono-adp-ribose-e6f6a-rabbit-mab/83732
PARP1 (46D11) Rabbit mAb #9532 (CST) validation stated on supplier's website: https://www.cellsignal.com/products/primary-antibodies/parp-46d11-rabbit-mab/9532
HPF1 Rabbit pAb HPA043467 (Atlas Antibodies) validation stated on supplier's website: https://www.atlasantibodies.com/products/antibodies/primary-antibodies/triple-a-polyclonals/hpf1-antibody-hpa043467/
ADPRHL2 (ARH3) Rabbit pAb HPA027104 (Atlas Antibodies) validation stated on supplier's website: https://www.atlasantibodies.com/products/antibodies/primary-antibodies/triple-a-polyclonals/adprhl2-antibody-hpa027104/
GAPDH Rabbit pAb ab9485 (Abcam) validation stated on supplier's website: https://www.abcam.com/gapdh-antibody-loading-control-ab9485.html
GFP Mouse mAb clone 7.1 and 13.1 (Roche) validation stated on supplier's website: https://www.sigmaaldrich.com/DK/en/product/roche/11814460001
Pan-ADPr (MABE1016) Rabbit (Millipore) validation stated on supplier's website: https://www.merckmillipore.com/DK/en/product/Anti-pan-ADP-ribose-binding-reagent,MM_NF-MABE1016
GFP (ab290) Rabbit (Abcam) validation stated on supplier's website: https://www.abcam.com/gfp-antibody-ab290.html
The custom-made anti-HPF1 antibody was validated by us with immunoblotting using purified human HPF1 and 293T and U2OS WT and HPF1 KO cells (Gibbs-Seymour et al., Mol Cell, 2016, Palazzo et al., eLife, 2018), as well as testis, ovary, brain, heart, kidney and liver tissue from WT and HPF1 KO mice (unpublished).
Goat-anti-rabbit HRP conjugated secondary antibody (Jackson Immunoresearch, 111-036-045) validation stated on supplier's website: https://www.jacksonimmuno.com/catalog/products/111-036-045
Goat-anti-mouse HRP conjugated secondary antibody (Jackson Immunoresearch, 115-036-062) validation stated on supplier's website: https://www.jacksonimmuno.com/catalog/products/115-036-062

Eukaryotic cell lines

Policy information about cell lines

Cell line source(s)	HeLa cells (CCL-2, female), U-2 OS (U2OS) cells (HTB-96, female), and HEK293T cells (CRL-3216, female), were acquired via the American Type Culture Collection.
Authentication	Cells were not routinely authenticated.
Mycoplasma contamination	All cells were tested for mycoplasma every two weeks, and no cells were contaminated.
Commonly misidentified lines (See ICLAC register)	No commonly misidentified cell lines were used in this study.
